# True cost estimation of common imaging procedures for cost-effectiveness analysis - insights from a Singapore hospital emergency department

**DOI:** 10.1016/j.ejro.2024.100605

**Published:** 2024-10-19

**Authors:** Yi Xiang Tay, Marcus EH Ong, Shane J. Foley, Robert Chun Chen, Lai Peng Chan, Ronan Killeen, May San Mak, Jonathan P. McNulty, Kularatna Sanjeewa

**Affiliations:** a Radiography Department, Allied Health Division, Singapore General Hospital,Outram Road, Singapore 169608, Singapore; b Radiography and Diagnostic Imaging, School of Medicine, University College Dublin, Belfield, Dublin, Ireland; c Duke-NUS Graduate Medical School, 8 College Road, Singapore 169857, Singapore; d Department of Emergency Medicine, Division of Medicine, Singapore General Hospital, Outram Road, Singapore 169608, Singapore; e Department of Neuroradiology, Division of Radiological Sciences, Singapore General Hospital, Outram Road, Singapore 169608, Singapore; f National Neuroscience Institute, 11 Jalan Tan Tock Seng, Singapore 308433, Singapore; g Department of Diagnostic Radiology, Division of Radiological Sciences, Singapore General Hospital, Outram Road, Singapore 169608, Singapore; h St Vincent's University Hospital, Elm Park, Dublin, Ireland; i School of Medicine, University College Dublin, Belfield, Dublin, Ireland

**Keywords:** Emergency Department, Cost, Diagnostic imaging, Cost savings, Cost-effectiveness analysis

## Abstract

**Objectives:**

There is a lack of clear and consistent cost reporting for cost-effectiveness analysis in radiology. Estimates are often obtained using costing derived from hospital charge records. This study aims to evaluate the accuracy of hospital charge records compared to a Singapore hospital's true diagnostic imaging costs.

**Methods:**

A seven-step process involving a bottom-up micro-costing approach was devised and followed to calculate the cost of imaging using actual data from a clinical setting. We retrieved electronic data from a random sample of 96 emergency department patients who had CT brain, CT and X-ray cervical spine, and X-ray lumbar spine performed to calculate the parameters required for cost estimation. We adjusted imaging duration and number of performing personnel to account for variations.

**Results:**

Our approach determined the average cost for the following imaging procedures: CT brain (€154.00), CT and X-ray cervical spine (€177.14 and €68.22), and X-ray lumbar spine (€79.85). We found that the true cost of both conventional radiography procedures was marginally higher than the subsidized patient charge, and all costs were slightly lower than the private patient charge except for X-ray lumbar spine (€73.49 vs.€79.85). We identified larger differences in cost for both CT procedures and smaller differences in cost for conventional radiography procedures, depending on the patient's private or subsidized status. For private status, the differences were: CT brain (Min: €194.20; Max: €264.40), CT cervical spine (Min: €219.54; Max: €399.05), X-ray cervical spine (Min: €5.27; Max: €61.94), and X-ray lumbar spine (Min: €6.36; Max: €108.04), while for subsidized status, the differences were: CT brain (Min: €7.56; Max: €62.64), CT cervical spine (Min: €47.02; Max: €132.49), X-ray cervical spine (Min: €15.88; Max: €103.44), and X-ray lumbar spine (Min: €13.66; Max: €149.44). Considering examination duration and the number of personnel engaged in a procedure, there were significant variations in the minimum, average, and maximum imaging costs.

**Conclusion:**

There is a modest gap between hospital charges and actual costs, and we must therefore exercise caution and recognize the limitations of utilizing hospital charge records as absolute metrics for cost-effectiveness analysis*.* Our detailed approach can potentially enable more accurate imaging cost determination for future studies.

## Highlights

• The hospital charge for imaging is not an accurate reflection of the true cost but is commonly used in cost-effectiveness analysis.
• A systematic method for determining costs has been devised, providing a breakdown of the costs associated with each component
• The use of hospital charge records as absolute metrics for cost-effectiveness analysis should be avoided.

## 1 Introduction

Healthcare facilities worldwide conduct approximately 3.6 billion imaging examinations every year [1]. Among them, around 84 % are conventional radiography (CR), 8 % are computed tomography (CT), and the rest consist of ultrasound, magnetic resonance imaging, and nuclear medicine studies [1]. The cost of these diagnostic imaging procedures account for around 10 % of healthcare expenses, and is projected to further increase against the backdrop of technological advancement, disease preventive efforts and defensive medicine [2], [3].

Despite its potential harm to patients, wastage of limited healthcare resources, and minimal or no impact on patient management, low-value imaging is widely prevalent, accounting for 20–50 % of all imaging internationally. [1]. Overuse of diagnostic imaging can lead to undesirable consequences. Patients may face significant physical and financial ramifications, including increased radiation exposure, adverse effects from intravenous contrast agents, and medical treatments or, procedures with uncertain clinical benefits [2]. The escalating costs associated with healthcare provision poses a pressing concern. Medical insurance premiums are increasing, and there is a rise in taxes to provide more funding for healthcare expenses [4], [5].

A recent systematic review indicates that the expense associated with low-value imaging may amount to billions of US dollars annually, emphasizing the need for additional actions to address this issue [1]. Indeed, several radiology societies have advocated the implementation of imaging referral guidelines and other interventions, such as clinical decision support systems, to promote value-based imaging [6], [7], [8]. In light of this situation, the radiology field is now required to establish practices that are cost-effective, promoting value-based radiology. Consequently, cost-effectiveness analysis (CEA) is gaining more prominence in the field of radiology [6].

CEA takes into account both direct and indirect costs [6]. The cost of imaging is a direct cost but also has indirect costs, depending on the perspective taken in the analysis. Estimating the ‘true’ cost associated with imaging procedures is important, ensuring that policymakers are well-informed when utilizing CEA findings. However, the results of a systematic review indicated that there was a lack of clear and consistent reporting of costs in the radiology setting, with different levels of data reported [6]. This led to the authors proposing the inclusion of a detailed explanation of cost calculation methodologies and sourcing in CEA publications.

Many authors use cost estimates derived from the literature or publicly available health service data [9]. However, it is important to note that these charges might vary significantly between hospitals and countries. This variation can result in either an underestimation or an overestimation of costs, affecting the accuracy and generalizability of locally conducted CEAs. Furthermore, hospital charge records may overstate actual costs, with substantial variations across different institutions [10]. This is seen in clinical trials, where the research rate for imaging conducted by the National Institutes of Health is considerably lower than the typical charge at most hospitals [9]. Therefore, the use of such quantum *‘represents an artificial estimate with little bearing on real-world economics’* [9]. Indeed, this emphasizes the importance of assessing the accuracy of hospital imaging charges, even without a universal or precise method of evaluating these costs [9].

Accurate costing requires a significant amount of time and effort, and it is also necessary to determine the level of accuracy and precision required for cost estimates [11]. There are multiple approaches to estimating costs; however, all methods must consider two key factors: the level of detail utilized in identifying and measuring resource and cost components and the method used to assign value to these components [11]. In addition, it is crucial to take into account the purpose and perspective of the study under consideration [11]. Together, these factors can assist in determining an appropriate costing methodology for the study such as bottom-up micro-costing, bottom-up gross-costing, top-down micro-costing, and top-down gross-costing.

In this study, we propose a cost calculation method for estimating the real cost of imaging. The main goal of our work is to evaluate and compare the ‘true’ cost of performing specific imaging procedures while drawing comparisons against hospital charge records (charge per procedure). We identified the emergency department (ED) as the study area due to the well-documented issue of low-value imaging in this setting [12]. In addition, as illustrated in systematic and scoping reviews, we focused on imaging of the brain (CT), cervical spine (CT/CR), and lumbar spine (CR), which are frequently associated with low-value imaging; likewise, CR and CT are the most commonly used imaging techniques [1], [13].

## 2 Materials and methods

The cost calculation method is a process that determines a cost estimate by taking into account direct costs (cost of a resource that is used by a single cost object, e.g. manpower cost), indirect costs (cost of a resource that is acquired to be used by more than one cost object but is a variable cost, e.g. housekeeping) and other related costs (expenses incurred at the organizational level, e.g. general overheads) [11]. Simultaneously, we will illustrate a cost breakdown structure to showcase all the costs linked to the imaging procedure. In this method, we will adopt a bottom-up micro-costing approach, providing a comprehensive breakdown of the costs associated with each individual component.

### 2.1 Current baseline estimates

The analysis takes place in the ED of a public hospital in Singapore, which is one of the busiest in the country. The current imaging costs for the identified imaging procedures are obtained from the hospital charge records. The ‘charge’ is the price the patient is billed for the service in the hospital and will serve as baseline estimates for comparison with the estimates from our methodology. Timestamps and personnel-related data were retrieved from the Radiology Information System by a member of the imaging informatics team. Microsoft Excel spreadsheet (Microsoft Corporation, Redmond, USA) was used to enter, store, analyze, and visualize the obtained data and values. The currency in the article has been standardized to Euros (€) in May 2024; however for practical reasons, Singapore dollars (S$) have been used in the initial data collection for this reporting [14].

The evaluation period was from January 2023 to December 2023.

### 2.2 Cost estimation method

The process followed for estimating the cost of CR and CT imaging consists of seven steps:

1. The total number of procedures performed per year for the identified imaging equipment (A).
2. Identification of equipment used for the procedure. These are fixed costs that originate from the CR or CT units which have a lengthy lifespan and are therefore spread out over numerous years through depreciation [15]. Examples of such parameters include capital expenditures, year of purchase, lifespan of imaging equipment, maintenance year and cost of preventative maintenance.

We estimated the total maintenance costs by considering the equipment's lifespan and the annual maintenance expenses. The maintenance duration was calculated by subtracting the warranty period from the equipment's lifespan. Therefore, the total maintenance cost of equipment was calculated by multiplying the maintenance duration by the annual maintenance cost.

The total equipment costs (B) for the lifespan of the imaging unit were estimated by considering the purchase price of the equipment and the overall maintenance cost. The equipment cost per procedure was determined by using the formula B / (A * lifespan of the equipment).

Identification of the manpower involved in the procedure. This step was further subdivided into radiographers and radiologists. It was essential to determine the average duration that each professional involved in the procedure spent - the time radiographers took to prepare the patient, perform the procedure (the routine projections for X-ray cervical spine and lumbar spine in the ED were Anterior Posterior and Lateral), and/or transfer the patient and the time used by radiologists to justify and/or report the imaging procedure. The manpower salary scale (blended hourly rate) was obtained as there were different grades or seniority of professionals involved and, therefore, a variation in costs for the manpower.

The duration of radiologist manpower (X) was determined by calculating the time the radiologist spent justifying and reporting the imaging procedure. Similarly, the time the radiographers spent on tasks such as transporting the patient, registering the patient, justifying, and performing procedures was measured (Y). Additionally, we gathered the time the healthcare attendant spent preparing the patient for the procedure (Z).

To this end, the time spent by various professionals during each procedure phase was obtained retrospectively from the radiology information system. This also encompasses the different grades or seniority of professionals involved. This data was collected from a convenience random sample of 96 patients (24 patients for each imaging procedure) who visited the ED in 2023.

The total manpower costs were estimated using the formula (X * manpower salary scale of radiologist) + (Y * manpower salary scale of radiographers) + (Z * manpower salary scale of healthcare attendant). Considering that each procedure can differ in manpower, complexity, and protocol, we have calculated the average time first and then estimated the average costs associated with the respective personnel.

To accommodate variations in imaging duration and the number of performing radiographers, we calculated the minimum and maximum overall manpower costs along with the average baseline. To calculate the minimum overall manpower costs, we determined the shortest duration required to complete the imaging procedure. We identified the combination of radiographers that resulted in the lowest hourly rate. Similarly, the combination of the longest imaging duration and highest hourly rate was used to estimate the maximum overall manpower costs.

1. Consumables used per procedure. Consumables/supplies like disinfectant wipes, gloves, and alcohol-based hand disinfectants were classified as variable costs. While there were data limitations for this parameter, we observed and approached practicing radiographers during the performance of the procedures to obtain the best estimate that reflects the current usage of these consumables for the respective imaging performed. The unit price of the consumables was obtained from the logistics team and used to calculate the total cost of consumables used during the procedure.
2. We endeavored to calculate the costs associated with patients waiting in the ED due to the imaging procedure. The total patient waiting time in the ED was the sum of the wait time from the creation of the imaging referral to the commencement of the imaging procedure and the wait time from the completion of the imaging procedure to the availability of the radiological report. We established the average total patient waiting time based on data from 96 patients, accounting for delays in reporting turnaround time that occurred after office hours by adjusting the values to the average office hours reporting turnaround time.

The Singapore government offers subsidies for observation ward stays, with the amount of payment determined by the monthly per capita household income bands [16]. To determine the subsidy amount for observation ward stays, we used the median monthly household income from work per household member to estimate the cost. Given that our study takes the hospital perspective, we estimated the observation ward’s cost by subtracting *the observation ward’s cost with a 50 % subsidy* from *the cost of the observation ward without any subsidy*. (Median household income in 2023 = S$3500; per capita household income > S$3100; 50 % subsidy) [17].

The cost of patient waiting was then estimated by multiplying the *cost of observation ward stay per hour by the total patient waiting time*.

1. Facility related allocated costs, which refers to the cost charged to the ED by the hospital for the use of floor space and facilities calculated from previous locally published literature [18] and estimates adjusted using CCEMG-EPPI Centre Cost Converter (v.1.7 last update: January 2024) [14] to account for the difference in price year.
2. The last step considers carbon emissions, energy consumption, and their corresponding costs. The equipment’s carbon emissions and energy consumption of the equipment were calculated using data obtained from the literature [19], [20], [21]. Prevailing carbon tax and electricity tariff was applied to the parameters to determine the associated costs. For carbon emissions calculation, the following formula was applied (kg CO2equivalent/scan * prevailing carbon tax), whereas, for energy consumption, the formula used was (energy consumption per examination * prevailing electricity tariff).

## 3 Results

At the study site, the estimated number of procedures performed by a single CR and single CT unit in a year was 34,500 and 16,500, respectively. The general departmental rules for the units were a lifetime of eight years, with the first two years covered by a warranty. Table 1 lists the associated imaging unit, maintenance, tariff, tax and consumables costs.

**Table 1:** Aggregate estimates for parameters used in the cost calculation.

Parameters	Cost, S$^a^
CR unit cost	495,000
CR unit preventive maintenance cost / year	11,000
CT unit cost	2,780,000
CT unit preventive maintenance cost / year	200,000
Disinfectant wipes	0.16
Gloves	0.14
Alcohol handrub	0.01
Zip lock bag	0.02
Incontinence pad	0.19
Denture cup	0.14
Carbon tax (per kg CO2e)	0.025
Electricity tariff (per kWh)	0.3258
Patient waiting in ED (per minute)	0.1089

a Costs in the Table are in Singapore dollars (S$), in May 2024, €1=S$1.45.

Manpower costs primarily account for the cost of performing an imaging procedure in the ED and range from 60.3 % to 72.6 % (Table 2). This was followed by facility related allocated costs (9.7–25.2 %), costs for patients waiting in the ED (Cost of ED crowding) (3.8–12 %) and equipment cost per procedure (1.8–13.4 %).

**Table 2:** Cost breakdown for conventional radiography (CR) and computed tomography (CT).

	Cervical Spine X-ray Anterior Posterior /Lateral	Lumbar Spine X-ray Anterior Posterior /Lateral	CT Brain	CT Cervical Spine
Cost Component	Cost, S$^a^	Cost, S$^a^	Cost, S$^a^	Cost, S$^a^
Equipment cost per procedure	2.06	2.06	29.91	29.91
Manpower cost for radiographers/radiologic technologists (Pre-procedure)	5.00	5.00	15.00	15.00
Manpower cost for radiographers/radiologic technologists (During procedure)	33.43	55.54	33.82	70.45
Manpower cost for healthcare attendant	2.50	2.50	Not Applicable	Not Applicable
Manpower cost for radiologist (reporting)	18.75	21.04	101.67	96
Consumables	0.31	0.31	0.33	0.66
Facility related allocated cost	24.93	24.93	24.93	24.93
Patient waiting in ED	11.87	4.35	17.20	19.38
Carbon emission	0.04	0.04	0.23	0.23
Energy consumption	0.02	0.02	0.22	0.29
Total	98.91	115.79	223.31	256.85

a Costs in the Table are in Singapore dollars (S$), in May 2024, €1=S$1.45.

The examination of variances in imaging duration (Table 3) and the number of personnel engaged in the procedure reveals significant variations in the minimum, average, and maximum cost associated with performing an imaging procedure.

**Table 3:** Duration, manpower count and patient wait time for imaging procedure in the ED.

	Mean (Min – Max)^b^ minutes*	Mean (Min – Max)^b^ *person*
X-ray Cervical Spine	18.3 (4.0 – 56.0)	1.5 (1.0 – 2.0)
X-ray Lumbar Spine	27.0 (6.0–76.0)	1.6 (1.0 – 2.0)
CT Brain	11.8 (6.0 – 22.0)	2.5 (2.0 – 4.0)
CT Cervical Spine	26.2 (8.0 – 108.0)	0.2(1.0 – 3.0)
	Wait Time for Imaging *minutes*	Report Turn-around Time *minutes*
	Median (IQR)^c^	Median (IQR)^c^
X-ray Cervical Spine	14.0 (6.0 – 26.8)	78.0 (34.8 – 498.8)
X-ray Lumbar Spine	10.0 (2.75 – 18.0)	45.5 (19.5 – 688.8)
CT Brain	19.0 (9.8 – 52.3)	104.5 (49.0 – 627.2)
CT Cervical Spine	8.5 (4.0 – 19.75)	40.0 (25.3 – 136.3)

b Min = minimum; Max = maximum

c IQR = interquartile range

* Extended duration of examination may be due to patients presenting for multiple imaging procedures (i.e. imaging protocol for trauma patients)

The costs for patients waiting in the ED, which are associated with the wait time for imaging and report turnaround time, vary across all four imaging procedures (Table 3). The report turn-around time (TAT) varies; however, it is within the predetermined ED TAT of 24 h (1440 min). The actual TAT depends on the referral category and whether the reporting is done during or after office hours.

According to the Ministry of Health, Singapore guidelines, patients are classified into two categories: subsidized and private. Overall, our cost calculation approach revealed that the average cost of three imaging procedures, excluding lumbar spine X-ray (€73.49 vs.€79.85), was lower than the typical bill size for patients who were in private class (Table 4). When examining patients who received subsidies (50 % subsidy), the cost of CR imaging procedures exceeded the typical bill size. However, there were still disparities in the cost of CT procedures, albeit narrowed. When we took into account the variations in manpower costs, we found that all imaging procedures had lower costs compared to the typical private hospital charge when we considered minimum manpower costs. However, all CR imaging procedures had higher costs than the typical hospital charge when maximum manpower costs were used for analysis.

**Table 4:** Summary of cost for performing imaging procedure in ED.

Imaging Procedure	Hospital Charge^d^ (Subsidized)	Hospital Charge (Private)	Our Method (Cost)	Difference between Cost & Charge (Subsidized)	Difference between Cost & Charge (Private)
X-ray Cervical Spine	€32.09	€73.49	Baseline average: €68.22	Baseline average: €36.13	Baseline average: -€5.27
			Min: €47.97	Min: €15.88	Min: -€25.52
			Max: €135.43	Max: €103.44	Max: €61.94

X-ray Lumbar Spine	€32.09	€73.49	Baseline average: €79.85	Baseline average: €47.76	Baseline average: €6.36
			Min: €45.75	Min: €13.66	Min: -€27.74
			Max: €181.53	Max: €149.44	Max: €108.04

CT Brain	€201.76	€403.52	Baseline average: €154.00	Baseline average: -€47.76	Baseline average: -€249.52
			Min: €139.12	Min: -€62.64	Min: -€264.40
			Max: €209.32	Max: €7.56	Max: -€194.20

CT Cervical Spine	€266.56	€533.12	Baseline average: €177.14	Baseline average: -€89.42	Baseline average: -€355.98
			Min: €134.07	Min: -€132.49	Min: -€399.05
			Max: €313.58	Max: €47.02	Max: -€219.54

^d^Based on 50 % subsidy (Median household income = S$3500; PCHI > S$3100; 50 % subsidy)

## 4 Discussion

This study illustrates a curated cost calculation method for estimating the cost of performing imaging in the ED for four common imaging procedures. Generally, our study revealed differences between the quantum of hospital charges and the estimates from our methodology, which closely reflect actual direct resource use and consumption.

Using true cost estimates derived from actual resource utilization is relevant for economic evaluations since it provides health policymakers with reliable cost information [22]. In tandem with that, the inclusion of comprehensive cost inventory tables that list baseline costs, sensitivity analysis ranges, the cost calculation methods and sourcing information can enhance the value of cost information while facilitating transparency and comparability across different studies [6]. However, a systematic review [6] has indicated that radiology publications featuring cost inventory tables exhibit varying levels of detail regarding inclusion of direct and indirect costs in their estimates. Unclear reporting has implications for the ability to replicate studies and difficulties for readers in interpreting the findings.

It was also suggested in the systematic review [6] that research primarily obtained cost data from Medicare reimbursements, international equivalents, hospital charge records, and costing manuals. However, the use of such data raises concerns. This is especially true for retrospective studies that analyze cost savings from the hospital's perspective, particularly when examining how the introduction of an intervention has altered the patient's trajectory [23]. An example is the cost savings arising from implementing imaging referral guidelines within the hospital. The retrospective analysis of the imaging referrals may suggest the potential avoidance of many imaging procedures. This would result in cost savings for the hospital, especially considering their transition to a capitation funding model (a model whereby healthcare providers receive a pre-determined fee for each resident living in the region they are caring for). However, these cost savings reflect cost avoidance (hospital charge), which means that by adhering to imaging referral guidelines, it has been established that the patient's trajectory (journey) has been altered [23]. Similarly, the hospital charge also does not accurately represent the exact sum of revenue a hospital receives for an imaging procedure [15]. Instead, the true extent of cost to the hospital is determined by the direct and indirect cost estimations of performing the imaging procedures.

Our study found that at least 60 % of the estimated cost of performing an imaging procedure was attributed to manpower when all four imaging procedures were examined. The manpower costs of radiologists reporting the CR procedures was lower than the manpower costs of radiographers performing the examinations. However, the manpower costs of radiologists reporting the CT procedures was greater than costs of the radiographer(s) time. This is mainly due to the difference in time to report CR (5 min) and CT (20 min) procedures. Indeed, reporting a CT procedure can take up to five times longer than reporting a CR procedure [24]. We applied varied imaging durations (captured time values) and numbers of performing staff to calculate total manpower costs for radiographer(s) to determine the average, maximum, and minimum costs associated with performing imaging. This approach aligns with other research, where the authors assert that the costs associated with manpower alone account for a substantial portion of the total cost, indicating a potential impact on overall costs [25].

In terms of patient wait time for imaging in the ED, our study findings were aligned with the literature [26]. While most procedures involved a single reader, there were some that required double reading, such as when the first reader was a junior and/or had no signing-off rights for radiological reports. Consequently, this impacted the TAT, which also affected the calculation of the costs of patients waiting in the ED. As a result, we adjusted the after-office TAT to the average during-office TAT. We inferred that residents may have already played a crucial role in pre-dictating cases or providing preliminary interpretations/reports for certain cases; as such, patients did not remain in the ED until the availability of the final radiological report, after-office hours. These practices are frequently observed in the ED radiology department, where specific strategies are implemented to provide high-quality patient care, regardless of the time of day [27].

A particularly interesting finding was the cost differences across the two imaging modalities (CT > CR). This relationship is likely due to a variety of factors. In our methodology, we considered the costs of purchasing the equipment, preventative maintenance, and the equipment's maintenance year and lifetime. While both imaging modalities have similar lifetimes, the significant disparity in equipment costs per procedure between CT (S$29.91) and CR (S$2.06) is primarily due to the higher purchase price and preventative maintenance expenses. Although the initial costs of acquiring the equipment are significant, the ongoing costs for maintenance and operation also constitute a substantial portion of the total equipment costs [28]. This increases CT's overall equipment costs. Furthermore, the equipment performs varying numbers of procedures per year (CR > CT), with CR having a higher throughput than CT. We can only speculate that the hospital charge (revenue margin) for CT is higher than CR due to the aforementioned factors and the relative operating expenses (variable cost) for CT.

Similarly, CT’s energy consumption and carbon footprint are larger than those of CR. Furthermore, it is worth noting that our approach considered energy consumption per procedure. However, existing literature has indicated that in the case of CT, about two-thirds of the energy consumption occurs when the system is in an idle state [20]. Although it is likely time-consuming and costly, it is important to consider this factor when calculating the cost [22].

Given the growing significance of CEA in healthcare decision-making, it is crucial to do high-value and clinically relevant research to inform health policymakers [29]. Cost is an essential component in CEA, as it directly affects the final result’s accuracy level, namely the incremental cost-effectiveness ratios. For instance, to determine the economic impact of a clinical decision support system in a clinical setting, it is necessary to include all pertinent cost components (infrastructure, licensing etc.) and actual cost savings (manpower, equipment usage etc.). The common practice of solely relying on the financial data reported before and after the intervention, which is typically calculated by multiplying the price per healthcare resource utilization by the total number of healthcare resources or services utilized, is not in accordance with the recommendations of the Consolidated Health Economic Evaluation Reporting Standards [30]. Therefore, its importance cannot be over emphasized [31]. Multiple authors have advocated the use of real costs in a CEA, which our methodology strives to promote [22], [31]. Indeed, it has been proposed that tariffs or reimbursement fees should only be utilized if they accurately reflect the actual costs [31]. Therefore, we calculated the costs based on real costs obtained from actual clinical settings. We also adjusted and estimated certain associated costs when data was lacking.

Future radiology research efforts may prioritize estimating indirect costs (overheads and capital costs) and assessing direct costs (repeat imaging), which correspond to the utilization of resources that are very closely associated with the patients responsible for incurring them. We aim to use the results of this study to undertake an economic evaluation in the near future to support the decision-making process for implementing hospital initiatives such as clinical decision support systems.

An important strength of our study is the involvement of a health economist in the evaluation team. According to evidence-based principles, the health economist conducted a comprehensive assessment of the variables to determine the inclusion of parameters likely to impact the study's findings. This ensured that our evaluation adhered to proper methods of performing and reporting analyses, addressing a concern raised in a previous systematic review [6] on cost-effectiveness analysis in radiology. In addition, we utilized actual time stamps and databases to gather data on multiple parameters. The timestamps data provided an opportunity to evaluate the trend in the patient waiting time, imaging duration and TAT. By accessing the database to retrieve information on the number of personnel and their specific job designations, it becomes plausible to estimate the costs associated with manpower in real-world clinical settings. Undoubtedly, when the cost component constitutes a significant portion of the total cost, it is imperative to allocate more attention to accurately identifying it [11]. In essence, having an overview of this information allows for adjusting certain variables that could affect the final computed costs.

### 4.1 Limitations

Despite the richness of the data, this evaluation had its limitations due to the unavailability of sensitive financial information, such as hospital overheads. Recognizing that many more variables contribute to the overhead, such as ancillary services, administrative supplies and services, and miscellaneous operational expenses (e.g., the Radiology Information System, Picture Archiving and Communication System, data storage, etc.), is crucial. We should not overlook this significant contribution to imaging costs since it contributes to the differences in hospital charges, which would be priced to recover such costs. This could be overcome by engaging and working closely with the hospital finance team to trace overheads (top-down costs). Additionally, the presented methodology also does not take into consideration the measurement of efficiency. Furthermore, the patient’s waiting time in the ED was an estimation based on data retrieved from Radiology Information System. We triage patients in our ED based on their level of medical urgency, known as patient acuity categories [32]. Consequently, the severity of their condition determines the priority they receive for medical attention, which might affect the wait time for imaging procedures and radiological reports. Due to the lack of monitoring of the patient's journey in the ED and the absence of the patient acuity categories of the patients being analyzed, the actual wait time for patients could vary. Another limitation of our study is the relatively small number of patients included in the analysis to determine the different parameters. Regrettably, this implies that the estimate may lack statistical precision [11].

## 5 Conclusion

In conclusion, the hospital charge for imaging is not an accurate reflection of the true cost to the hospital and should not be used for cost-effectiveness calculations. While our cost calculation method does not include certain costs, such as overheads associated with ancillary services and administrative supplies and services, it may be a strong alternative as it provides a systematic and transparent method for determining costs.

## Ethics approval

Ethics approval is not required as this study is categorised a Service Evaluation.

## Funding

This work was supported by the SingHealth Talent Development Fund and Singapore General Hospital Scholarship.

## CRediT authorship contribution statement

**Yi Xiang Tay:** Writing – original draft, Methodology, Investigation, Formal analysis, Conceptualization. **Marcus EH Ong:** Writing – review & editing, Resources. **Shane J. Foley:** Writing – review & editing, Visualization. **Robert Chun Chen:** Writing – review & editing, Validation. **Lai Peng Chan:** Writing – review & editing, Validation. **Ronan Killeen:** Writing – review & editing. **May San Mak:** Writing – review & editing. **Jonathan P McNulty:** Writing – review & editing, Supervision, Conceptualization. **Kularatna Sanjeewa:** Writing – review & editing, Supervision, Methodology.

## Declaration of Competing Interest

The authors declare the following financial interests/personal relationships which may be considered as potential competing interests: Yi Xiang Tay reports financial support was provided by SingHealth Group. Yi Xiang Tay reports financial support was provided by Singapore General Hospital. Ronan Killeen reports a relationship with xWave Technologies Ltd that includes: consulting or advisory and equity or stocks. If there are other authors, they declare that they have no known competing financial interests or personal relationships that could have appeared to influence the work reported in this paper.

## Data Availability

The data that support the findings of this study are available from the corresponding author, [YX], upon reasonable request.
